# Involvement of peroxisome proliferator-activated receptors in the estradiol production of ovine Sertoli cells

**Published:** 2017-09-15

**Authors:** Hossein Hassanpour, Valiallah Khalaji-Pirbalouty, Manoochehr Adibi, Hassan Nazari

**Affiliations:** 1 Department of Gamete and Cloning, Research Institute of Animal Embryo Technology, Shahrekord University, Shahrekord, Iran;; 2 Department of Biology, Faculty of Science, Shahrekord University, Shahrekord, Iran.

**Keywords:** Aromatase, Estradiol, PPAR, Sertoli cell, Sheep

## Abstract

Peroxisome proliferator-activated receptors (PPARs) are nuclear receptors of transcription factors composed of three family members: PPARα, PPARβ/δ and PPARγ. This study was aimed to evaluate the role of PPARs in the estradiol production via follicle stimulating hormone (FSH) in the ovine Sertoli cells. At the first step, transcripts of PPARα, PPARβ /δ and PPARγ were evaluated by quantitative real time PCR (qRT-PCR) in the ovine Sertoli cells *in vitro* after FSH treatment. PPARγ transcript was increased in FSH-treated cells while PPARα and PPAR β /δ transcripts were unchanged. At the second step, Pioglitazone as PPARγ agonist and 2-chloro-5-nitrobenzanilide (GW9662) as PPARγ antagonist were used in the FSH-treated Sertoli cells and then, the estradiol production and aromatase transcript were evaluated. Aromatase transcript was increased by pioglitazone in the FSH-treated Sertoli cells while GW9662 did not change its transcript. The estradiol production was increased by low concentrations of pioglitazone in FSH-treated Sertoli cells while the production of this hormone was decreased by the high concentration of Pioglitazone. The GW9662 did not change the production of estradiol in FSH-treated Sertoli cells. It is concluded that FSH regulates the estradiol production and aromatase expression in a way independently of PPARβ/δ and PPARα activation, although FSH increases the transcript of PPARγ and in this way, it could affect (mostly increase) aromatase transcript and estradiol production. Probably, this effect of FSH in the estradiol production via PPARγ is only a servo-assist mechanism which if it was inhibited, the estradiol production was not considerably affected.

## Introduction

Peroxisome proliferator-activated receptors (PPARs) as nuclear receptors of transcription factors have three family members consist of PPARα, PPARβ/δ and PPARγ.^1^ Since the discovery of PPARs in 1990, numerous functions such as enhancing insulin sensitivity, regulating fat mass and cell proliferation as well as regulating inflammatory reactions have been attributed to these receptors.^2^ The PPARα regulates the expression of genes involved in fatty acid beta-oxidation to balance the energy homeostasis while PPARβ can down-regulate the inflammatory signaling.^3^ The PPARγ contribute to storage of fatty acids and to control the glucose metabolism, and has been implied in the pathogenesis of several diseases such as obesity and diabetes.^4^^,^^5^ These receptors are activated by binding of natural ligands, such as polyunsaturated fatty acids or by synthetic ligands. Synthetic molecules of the glitazone family are currently used to treat type II diabetes and also to attenuate the secondary clinical symptoms associated with insulin resistance, including polycystic ovary syndrome.^6^ The PPARs are expressed in the central nervous system and in the following reproduction tissues: gonads (ovary and testis), uterus, prostate, mammary and pituitary glands. Previous studies demonstrated that the three PPAR isoforms are expressed in the spermatozoa, interstitial Leydig cells, and seminiferous tubule cells (Sertoli and germ cells) of the testis.^7^^-^^9^

Male fertility and the process of spermatogenesis are dependent upon the somatic Sertoli cells to produce factors that are required for developing germ cells. Sertoli cells provide a specialized, protected environment within the seminiferous tubules of the testis for germ cell development.^10^ Sertoli cells produce factors required to fuel germ cell metabolism (lactate, transferrin, androgen binding protein), growth regulatory factors (Stem cell factor, SCF; Transforming growth factors, TGF-α and TGF-β), insulin-like growth factor-I (IGF-I), fibroblast growth factor (FGF), epidermal growth factor (EGF) and hormones that regulate the development of the male reproductive structures or feedback to regulate the hormonal signals affecting Sertoli cells (estrogen, mullerian inhibiting substance, and inhibin).^10^^,^^11^ Within the seminiferous tubules only Sertoli cells possess receptors for testosterone and follicle stimulating hormone (FSH) and thus these cells are the major targets of the ultimate hormonal signals that regulate spermatogenesis.^10^ A review study identified FSH-inducible genes in Sertoli cells including PPARs.^2^ On the other hand, it has been suggested that PPARs may contribute in the steroidogenesis.^2^ The objective of this study is to evaluate the effect of FSH in the expression of PPARα, PPARβ and PPARγ genes and to find which of these receptors could influence the gene expression of aromatase and estradiol production.

## Materials and Methods

**Cell cultures and treatments. **All materials used in this study, except those mentioned, were purchased from Sigma-Aldrich Co. (St. Louis, USA). In each following experiments, testes of 10 lambs (3 to 10 month-old) were collected from an abattoir, placed on ice and transferred to the laboratory within 1 hr. Sertoli cells were isolated from these testes and cultured according to Izadyar *et al*.^12^ and Hassanpour *et al*.^13^ Briefly, testes were decapsulated and minced into small pieces. For enzymatic digestion, testis pieces were suspended in Eagles minimum essential medium (EMEM) + 25 mM NaHCO_3_ and incubated for 1 hr at 37˚C with 50 IU mL^-1 ^DNase (Roche Diagnostics, Indianapolis, USA), 1 mg mL^-1 ^trypsin and 1 mg mL^-1 ^type IV collagenase (Gibco, Grand Island, USA). The samples were centrifuged at 400 *g* for 4 min and supernatants (containing Leydig cells) were discarded. In the next stage of enzymatic digestion, the samples were suspended in EMEM (Gibco) + 25 mM NaHCO_3_ and incubated at 37 ˚C (5% CO_2_) for 45 min with DNase (50 IU mL^-1^) and type IV collagenase (1 mg mL^-1^). The centrifugation was repeated again at 60 *g* for 30 sec. The supernatant was isolated, transferred to a fresh tube and then was centrifuged at 400 *g* for 4 min. The pooled cells were incubated at 32˚C in EMEM supplemented with 10% fetal calf serum (FCS; Gibco), 25 mmol NaHCO_3_, 2 mmol L-glutamine, 1% non-essential amino acids, 200 IU mL^-1 ^penicillin, 0.2 mg mL^-1 ^streptomycin and 15 mmol 4-(2-hydroxyethyl)-1-piperazineëthanesulfonic acid (HEPES) (Sigma-Aldrich Co.). After 24 hr, cells in the supernatant fluid were discarded. At this time, the round to cuboidal cells that adhere to the Petri dish are Sertoli cells. The culture process was continued with renewing the culture medium. After three passages, Sertoli cells with 70% confluency were used for treatments in each experiment. To characterize Sertoli cells, vimentin was detected in cultured Sertoli cells by immunocytochemistry.^14^^,^^15^

In the first experiment, FSH was added into the culture medium of Sertoli cells at concentrations of 0 (as control) and 0.2 IU mL^-1^ (selected from a primary dose–response study with a range of 0.1 to 0.4 IU mL^-1^ FSH). After the 72 hr of culture at 37 ˚C in a humidified atmosphere of 5% CO2 in the air, the sample of cultured Sertoli cells was centrifuged and then the upper liquid was transferred to a tube for measurement of estradiol. The pellet (containing Sertoli cells) was also stored at –70 ˚C for subsequent RNA extraction, cDNA synthesis and quantitative real time PCR (qRT-PCR). In this experiment, the relative gene expression of PPARα, PPARβ, PPARγ and aromatase were evaluated between control and FSH-treated groups.

The second experiment was designed based on the results of the first experiment. In this experiment, FSH was added to all samples of cultured Sertoli cells. In addition, GW9662 as PPARγ antagonist was used in three concentrations of 1, 5 or 10 µM. In another set of groups, Pioglitazone as PPARγ agonist was used in three concentrations of 1, 10 or 100 µM. After 72 hr of culture at 37 ˚C in a humidified atmosphere of 5% CO2 in the air, the sample of cultured Sertoli cells was centrifuged and then the upper liquid was transferred to a tube for measure-ment of estradiol. The pellet (containing Sertoli cells) was also stored at −70˚C for subsequent RNA extraction, cDNA synthesis and qRT-PCR. In this experiment, the relative gene expression of aromatase was evaluated between different concentrations of GW9662 or Pioglitazone.

**RNA extraction and cDNA synthesis. **In two experiments, total RNA from frozen Sertoli cells was extracted using RNXPlus reagent (Sinaclon Bioscience, Karaj, Iran). Homogenized cells were prepared in this solution, then were mixed with chloroform. The resulting mixture was centrifuged (9000 rpm, 4 ˚C, 15 min), yielding an upper aqueous phase containing total RNA. Following 100% isopropanol precipitation, the RNA pellet was washed with 75% ethanol. The RNA samples were re-suspended in diethylpyrocarbonate-treated water. Total RNA was treated with RNase-free DNase (Sinaclon Bioscience) to avoid amplification of contaminating genomic DNA. RNA was evaluated by agarose gel (1.5%) electrophoresis to determine extracted RNA quality as indicated by discrete 18S and 28S rRNA bands. The amount and quality of RNA were determined by spectrophotometry. Only RNAs of sufficient purity, having an absorbance ratio (A260/280) between 1.8 and 2.0, were considered for the synthesis of cDNA.^16^

Total RNA was immediately reverse transcribed into cDNA using M-MLV reverse transcriptase (Sinaclon Bioscience) as described by Hassanpour *et al*.^17^ The reverse-transcription (RT) was performed in a 20 µL reaction volume containing 2 µg of extracted RNA, 200 ng random hexamer, 0.5 mM dNTP. This mixture was heated to 65 ˚C for 5 min, and after incubation, 40 U of RNase inhibitor, 2 µL RT buffer (50 mM Tris-HCl, 75 mM KCl, 3 mM MgCl_2_), 10 mM DTT and 200 U M-MLV reverse transcriptase were added. This mixture was incubated for 5 min at 25 ˚C, followed by 50 min at 38 ˚C. The reverse transcription mix was heated to 75 ˚C for 15 min to denature the RNA and then stored at – 20 ˚C.

**Quantitative real-time PCR. **In this study, qRT-PCR was used to measure the changes in gene expression. The levels of PPARα, PPARβ, PPARγ, aromatase and Glyceraldehyde-3-phosphate dehydrogenase (GAPDH) transcripts were determined by qRT-PCR using SYBR^® ^Premix Ex Taq™II (Takara Bio Inc., Otsu, Japan). This method requires a suitable internal standard to control for variability between samples and to normalize the input load of cDNA. GAPDH gene as an endogenous standard was applied to normalize the input load of cDNA among samples. The GAPDH was found as the most stable housekeeping gene (confirmed by NormFinder software, Molecular Diagnostic Laboratory, Aarhus University Hospital Skejby, Denmark) in the ovine Sertoli cells during this study. Specific primers of PPARα, PPARβ, PPARγ, aromatase and GAPDH were designed with Primer-Blast.^18^ The expected products of primers in PCR were checked in Nucleotide-Blast which found no similarity with other ovine genes.^19^ Details of the primers are presented in Table 1. 

**Table 1:** Primers used for quantitative real time PCR analysis of ovine mRNAs

**Target**	**Primers**	**PCR product**	**Accession No.**
**GAPDH**	5'-GTTCCACGGCACAGTCAAGG-3' 5'- ACTCAGCACCAGCATCACCC-3'	117 bp	NM_001190390.1
**Aromatase**	5'-CCCTTCTGAGACGCTTCCAC-3' 5'- GGTCTCGTCTGGATGCAAGG-3'	95 bp	AJ012153.1
**PPARα**	5'-AGAACAAGGAAGCGGAAGTC-3' 5'-ATCCCGTCTTTGTTCATCAC-3'	199 bp	XM_004007050.1
**PPARβ**	5'-CAACGAGGGGAGTCAGCACA-3' 5'-AAGGGACTCCCAGCCGTTTG-3'	153 bp	XM_004018769.1
**PPARγ**	5'-GAGGGCGATCTTGACGGGAA-3' 5'-ACCTCTTTGCTGGGCTCCTG-3'	132 bp	NM_001100921.1

The PCRs were carried out in a real-time thermocycler (Rotor Gene Q 6000; Qiagen, Valencia, USA) in three replicates for each sample. The cDNA (1 µL) was added to the 10 µL of SYBR^®^ Premix Ex Taq II Mix and 0.75 µM of each specific primer in a total volume of 20 µL. The PCR program was 40 cycles of 94 ˚C for 40 sec, 65 ˚C for 35 sec and 72 ˚C for 30 sec. At the end of each phase, the amount of fluorescence was measured to quantitate objectives. The no-template control and no-reverse transcriptase control were used to check contamination in the PCR reagents. Gene expression data were normalized to GAPDH (as internal reference gene) and presented as gene/GAPDH. Data were analyzed using LinRegPCR software version 2012.0 (Experimental and Molecular Cardiology Group, Academic Medical Centre, University of Amsterdam, Amsterdam, The Netherlands), to give the threshold cycle (Ct) number. Mean efficiency values (E) for each gene were also determined from the amplification profiles of individual samples with this software.^20^ The mRNA level of each target gene relative to GAPDH was estimated for each sample in two experimental groups using efficiency (E) in the formula E _GAPDH _^(Ct sample) ^/ E _target _^(Ct sample)^. Then, the attained data were statistically analyzed. To determine fold change for each gene, the relative gene expression of the treated group relative to the control group were calculated as follows.^21^^,^^22^

*Ratio= (E *_GAPDH _^(Ct sample)^ */ E *_target _^(Ct sample)^ *):( E *_GAPDH _^(Ct control)^ */ E *_target _^(Ct control)^ *)*

**Estradiol measurement. **The amount of estradiol was measured in the medium of cultured Sertoli cells by Sheep Estradiol ELISA kit (Cusabio Biotech, Wuhan, China). The data were expressed as pg µg^-1^ total protein of cell cultures. Mass protein of cells was measured by Bradford method. 

**Statistical analysis. **All data points are presented as means ± SE. Differences between experimental group means were analyzed through paired *t *- test or one-way ANOVA followed by Duncan’s multiple range tests. Differences were considered significant at *p* < 0.05.

## Results

**Fig. 1:**
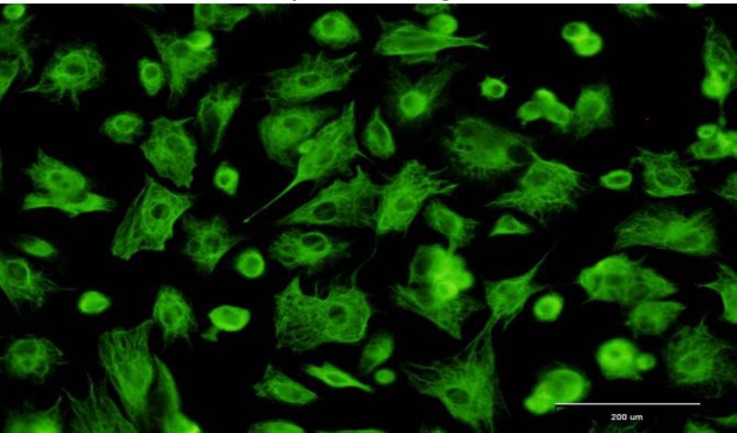
Microscopic morphology of Sertoli cells derived from 3 to 10 month-old lambs. Sertoli cells were characterized by immunocytochemistry. Sertoli cells were positive for Vimentin (green color), Bar = 200µm

**Ovine Sertoli cells isolation and characterization. **The presence of Vimentin protein on ovine Sertoli cells was confirmed by immunocytochemistry, demonstrating that the majority (up 90%) of freshly isolated cells were Vimentin positive (Fig. 1). All these results indicated that the Sertoli cells were properly isolated.

 **Estradiol production in Sertoli cells after exposing to FSH. **The amount of estradiol was measured in the culture medium of ovine Sertoli cells after adding of FSH. The amount of estradiol was significantly increased in the medium of the FSH-treated Sertoli cells grouo (12.90 ± 1.22 pg µg^-1^ cellular protein) compared to the control group (4.10 ± 0.88 pg µg^-1^ cellular protein), (*p* < 0.05).

**Fig. 2:**
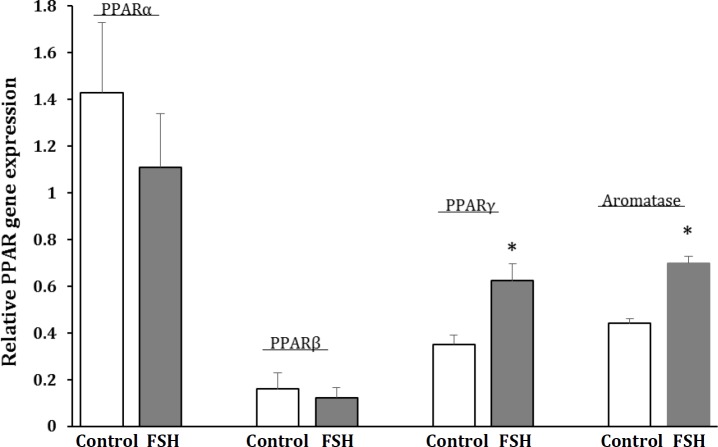
Relative expression of PPAR genes in ovine Sertoli cells after treated by FSH. Values are means ± SEM. * indicates significant difference compared to the control group at* p *< 0.05

**Relative expression of PPARs and aromatase genes in Sertoli cells after exposing to FSH. **Expression of PPARα, PPARβ, PPARγ and aromatase genes was studied using quantitative RT-PCR in the cultured Sertoli cells exposing to FSH. The results of qRT-PCR are shown in Fig. 2. Expression of PPARγ and aromatase genes were significantly increased (*p* < 0.05) about 1.8-fold and 1.6-fold respectively in the FSH-treated Sertoli cells compared to their controls while expression of PPARα and PPARβ were statistically unchanged. 

**Fig. 3:**
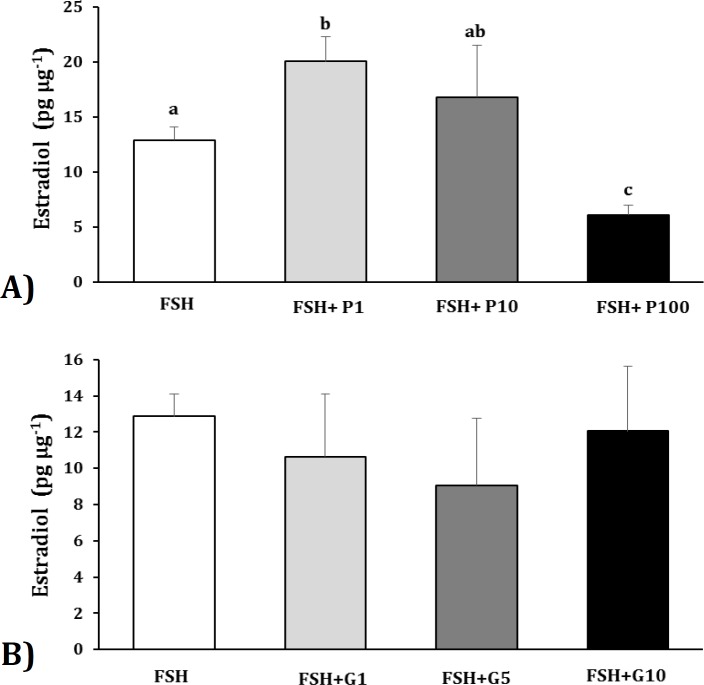
Effect of FSH and (A) Pioglitazone (P, PPARγ agonist) or (B) GW9662 (PPARγ antagonist) on estradiol production of ovine Sertoli cells. P1 (pioglitazone, concentration of 1 µM); P10 (pioglitazone, concentration of 10 µM); P100 (pioglitazone, concentration of 100 µM). G1 (GW9662, concentration of 1 µM); G5 (GW9662, concentration of 5 µM); G10 (GW9662, con-centration of 10 µM). Values are means ± SEM. ^abc^ Different letters indicate significant differences among the groups (*p *< 0.05

**Estradiol production in Sertoli cells after exposing to FSH and pioglitazone or GW9662. **The amount of estradiol was measured in the culture medium of ovine Sertoli cells after adding FSH and Pioglitazone (concentrations of 1, 10 and 100 µM), (Fig. 3A). The amount of estradiol was significantly increased by the concentration of 1 µM Pioglitazone compared to the control group while the estradiol was significantly decreased by the concentration of 100 µM Pioglitazone (*p* < 0.05). The production of this hormone did not differ between groups of control and treated by 10 µM Pioglitazone.

The amount of estradiol was also measured in the culture medium of Sertoli cells after adding of FSH and GW9662 (concentrations of 1, 5 and 10 µM), (Fig. 3B). The amount of this hormone was not differed significantly among different groups of the experiment. 

**Fig. 4:**
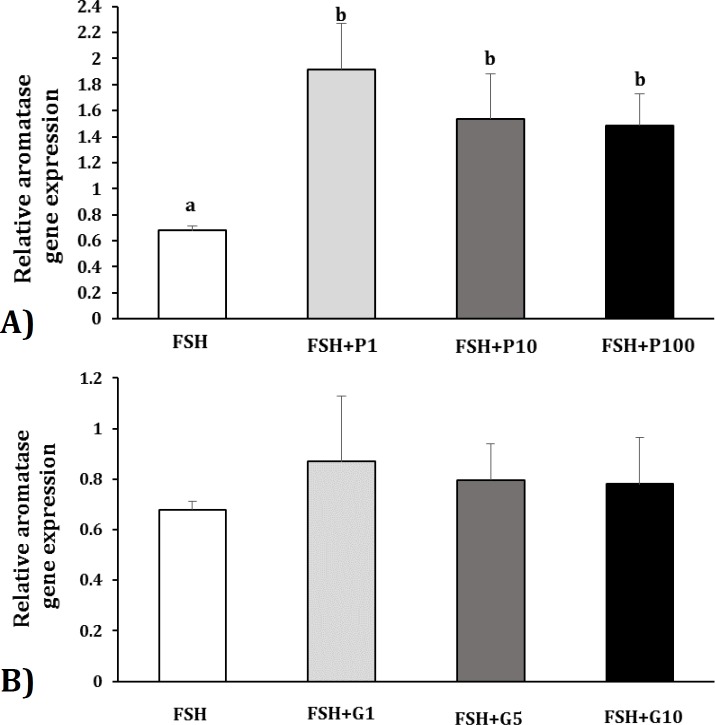
Relative expression of aromatase gene in ovine Sertoli cells after treatment by FSH and (A) Pioglitazone (PPARγ agonist) or (B) GW9662 (PPARγ antagonist). P1 (Pioglitazone, concentration of 1 µM); P10 (Pioglitazone, concentration of 10 µM); P100 (Pio-glitazone, concentration of 100 µM). G1 (GW9662, concentration of 1 µM); G5 (GW9662, concentration of 5 µM); G10 (GW9662, concentration of 10 µM). Values are means ± SEM. ^ab^ Different letters indicate significant differences among groups (*p *< 0.05

**Relative expression of aromatase gene in Sertoli cells after exposing to FSH and pioglitazone or GW9662. **Expression of aromatase gene was evaluated using qRT-PCR in the cultured Sertoli cells exposing to FSH and Pioglitazone or GW9662. The results of qRT-PCR are shown in Fig. 4. Expression of aromatase gene was significantly (*p* < 0.05) increased about 2.8-fold, 2.3-fold and 2.2-fold at the concentrations of 1, 10 and 100 µM of Pioglitazone respectively compared to the control group (Fig. 4A) while expression of this gene was not significantly differed by the concentrations of 1, 5 and 10 µM of GW9662 compared to the control group (Fig. 4B).

## Discussion

This study was aimed to examine the role of PPARs in estradiol production via FSH in the ovine Sertoli cells. FSH, a glycoprotein hormone secreted by the pituitary gland, is required for normal function and proliferation of Sertoli cells. FSH involves in the steroidogenesis via activation of phosphatidylinositol 3-kinase/protein kinase B (AKT) signaling pathway and subsequent stimulation of aromatase expression (gene and protein).^23^ The FSH also could stimulate the production of prostaglandins such as prostaglandin J2 (PGJ2) in the Sertoli cells.^24^ It has been reported that PGJ2 acts as a natural ligand of PPARγ in cells.^25^

In the present study, the expression of aromatase gene and estradiol production were assessed as an index involving FSH in the steroidogenesis. The expression of PPARs genes were also evaluated after FSH treatment in the Sertoli cells. The presence of PPARα, PPARβ and PPARγ mRNAs previously had been confirmed in the testis of human and rat,^7^^,^^8^^,^^26^^,^^27^ and it was demonstrated that FSH could stimulate the expression of PPARα gene in the testis and could contribute in the steroidogenesis via this receptor. Our study showed conversely that FSH could not affect the expression of PPARα and PPARβ genes in the cultured Sertoli cells of sheep, while the expression of PPARγ gene was increased by FSH. However, this conflicting effect of FSH on PPARs gene expression may be due to the use of different animals and different status of cells in the other studies.^7^^,^^8^^,^^26^^,^^27^ This condition has also been well reported in the studies of the function of these nucleus receptors in different animals.^2^ Activation of PPARγ and regulation of gene transcription is a multistep process that involves ligand binding, heterodimerisation with Retinoid X receptor (RXR), interaction with sequence-specific gene promoter elements, and recruitment of co-activators and other nuclear coregulatory proteins.^2^ Previous studies have shown that PPARγ agonists showed contradictory actions on the secretion of steroids (inhibition or stimulation of progesterone and estradiol production) in the human, rat, ovine, bovine and porcine granulosa cells.^26^^,^^28^^-^^33^ In the current study, Pioglitazone as PPARγ agonist was added in the culture of Sertoli cells. This treatment stimulated Sertoli cells to express high numbers of aromatase mRNA transcripts and sub-sequently to produce more estradiol. Although, the high concentration of this agonist decreased the production of estradiol, probably because of its apoptotic effect in the cells without affecting DNA function.^34^ The conflict function of Pioglitazone in the estradiol production has been reported by previous studies in the different cells and different animals. Hara *et al*. demonstrated that Pioglitazone counteracts the tumor necrosis factor-α inhibition of follicle-stimulating hormone-induced follicular development and estradiol production in an *in vitro* mouse preantral follicle culture system.^6^ On the other hand, Subbaramaiah *et al*. indicated that Pioglitazone suppresses Prostaglandin E2 leading to the reduced levels of aromatase and estradiol production in human mammary gland.^32^ However, our results about the action of PPARγ agonist in the ovine Sertoli cells was agreed with its function in the steroidogenesis in the rat and ovine granulosa cells reported previously*.*^26^^,^^31^

The GW9662 as a potent irreversible and selective PPARγ antagonist was used in the present study which did not showed considerable effect on the expression of aromatase gene and production of estradiol in FSH-treated Sertoli cells.^25^^,^^35^ Sharma and Singh added GW9662 + FSH to the culture of buffalo granulosa cells and found that this PPARγ antagonist does not change aromatase gene expression and estradiol production.^35^ The reason for this action of GW9662 is unclear while this PPARγ antagonist was expected to inhibit aromatase expression and estradiol production as opposite action of Pioglitazone. These actions of GW9662 have also been observed by Seargent *et al.* in the mammary gland cells.^36^ They suggested that GW9662, in addition to inhibition of PPARγ activity, may activate signal pathways independently from PPARγ which probably compensate the inhibition of PPARγ activity. It is probable that the PPARγ plays the role of servo-assist mechanism in the estradiol production because stimulation of this receptor increased the estradiol production but inhibiting this receptor did not change the steroidogenesis. 

It was concluded that FSH regulated the estradiol production and aromatase expression in an independent way from PPARβ/δ and PPARα activation, although FSH increased the expression of PPARγ gene and in this way it possibly affected (mostly increase) aromatase gene expression and estradiol production. Probably, this effect of FSH in the estradiol production via PPARγ is a servo-assist mechanism which if it is inhibited, the estradiol production may not be considerably affected. 
